# Enhanced Osteoinduction, Rheological and Mechanical Performance of 3D-Printed Methylcellulose-Gelatin-Hydroxyapatite Scaffolds

**DOI:** 10.3390/jfb17090443

**Published:** 2026-09-02

**Authors:** Ceren Yuksel, Simon Kwoon-Ho Chow, Ryota Hirose, Mayu Morita, Qi Gao, Takahiro Igei, Monica Thukkaram, Chao Ma, Tony Tam, Sophie Clarke, Mark Skylar Scott, Stuart Goodman, Duygu Ege

**Affiliations:** 1 Institute of Biomedical Engineering, Boğaziçi University, Istanbul 34684, Türkiye; ceren.yuksel@boun.edu.tr; 2 Department of Orthopaedic Surgery, Stanford University School of Medicine, Stanford, CA 94305, USA; 3 Department of Bioengineering, Stanford University School of Medicine, Stanford, CA 94305, USA; 4 Basic Science and Engineering Initiative, Children’s Heart Center, Stanford, CA 94304, USA; 5 Chan Zuckerberg Biohub, San Francisco, CA 94158, USA; 6 Institute for Stem Cell Biology and Regenerative Medicine, Stanford, CA 94305, USA; 7 Stanford Cardiovascular Institute, Department of Medicine, Stanford, CA 94305, USA

**Keywords:** 3D printing, bone tissue engineering, gelatin, hydroxyapatite, methylcellulose, osteogenic differentiation

## Abstract

Complex patient-specific bone defects remain difficult to reconstruct because the regenerative capacity of bone is limited and prefabricated implants cannot readily match defect geometry. In this work, methylcellulose-gelatin-hydroxyapatite (MC/GEL/HA) inks were formulated with varying methylcellulose content and hydroxyapatite incorporation, crosslinked with EDC/NHS, and 3D-printed into porous scaffolds with defined square-pore architectures. Inks were evaluated by oscillatory and steady-shear rheology, and printed scaffolds were characterized for morphology, chemical composition, mechanical performance, physicochemical stability, wettability, apatite-forming bioactivity, and osteogenic responses of human bone marrow mesenchymal stem cells. Methylcellulose content primarily governed ink rheology and printability, and increased compressive strength (up to ~0.38 MPa for 15MC/10GEL/30HA), whereas hydroxyapatite enhanced surface hydrophilicity, promoted apatite nucleation within 7 days in simulated body fluid, and markedly increased alkaline phosphatase activity (>10-fold over HA-free scaffolds), mineralization (~2-fold by Alizarin Red), and the highest osteocalcin expression among the HA-containing formulations (~2.45-fold at day 14). The 15MC/10GEL/30HA formulation showed the most favorable balance of printability, mechanical performance, and osteogenic performance. These complementary functions reconciled structural stability with osteogenic performance, supporting the MC/GEL/HA system as a tunable bioink platform for non-weight-bearing bone regeneration, while warranting further in vivo validation.

## 1. Introduction

Bone is a hierarchically organized connective tissue that fulfills critical structural and physiological roles in vertebrates. However, it remains susceptible to a range of injuries, including trauma, fractures, and tumor-related pathologies [1,2]. Autografts and allografts have long been the clinical gold standard, but each has inherent limitations. Autologous bone remains the preferred choice because it combines the three requirements of repair in a single graft: osteogenic cells, osteoinductive factors, and an osteoconductive matrix. However, its supply is limited, and harvesting may cause donor-site morbidity. Allografts avoid harvesting from the patient but bring their own drawbacks: the processing that reduces antigenicity also removes much of their osteoinductive potency, while some immunogenicity and a small risk of disease transmission remain [3]. These limitations have motivated the development of engineered substitutes that can be produced on demand and customized to each bone defect.

Additive manufacturing is particularly well suited to addressing the geometric aspect of this challenge. Skeletal defects are geometrically irregular, so prefabricated implants rarely conform to them and leave interfacial gaps that impair integration. Extrusion-based three-dimensional printing builds a construct layer by layer from a computer-aided design, giving precise, reproducible control over pore size, lattice geometry, and overall shape [1,2], which in turn improves cell infiltration and nutrient flow [4,5]. This enables patient-specific fabrication, particularly for geometrically complex non-weight-bearing defects such as those of the craniofacial skeleton.

The performance of a printed scaffold is largely determined by the choice of ink. Hydrogels emerge as an especially promising choice because of their hydrated, customizable network structure, mimicking properties of the extracellular matrix and capability of providing cells with a water-rich environment [6,7]. Gelatin (GEL), a denatured protein derived from collagen, maintains an Arg-Gly-Asp (RGD) tripeptide recognized by integrins, thus providing natural cell adhesive ability [8]. Nonetheless, gelatin suffers from considerable printability drawbacks as a result of its low viscosity causing sagging of filaments during extrusion, and thermoreversible gelation resulting in dissolution of printed constructs at physiological temperatures [9]. Methylcellulose (MC), a water-soluble cellulose derivative that carries hydrophobic methoxy groups, is extensively used for solving these problems through an increase in ink viscosity and introduction of thermoresponsive rheology, allowing for filament extrusion and self-support during the printing process [10]. Previous studies showed that the addition of MC improves printability and mechanical stability of gelatin-containing inks while maintaining biological compatibility [11,12,13,14]. For example, addition of 8% of MC to 5% GelMA raised the compressive modulus roughly 3-fold while osteoblasts encapsulated in this material remained viable with efficiency above 90% [11]. Hence, these results suggest that MC is the main ingredient responsible for printability, while gelatin provides a bioactive matrix containing RGD peptides for cell adhesion.

Despite these advances, the MC/GEL system still lacks the osteoconductive mineral component needed for active bone regeneration. Hydroxyapatite (HA), a calcium phosphate mineral comprising a major part of the inorganic fraction of bone, is osteoconductive and has direct bonding with surrounding bone, thus reinforcing the surrounding polymer [15]. While inherent brittleness and the slow resorption rate limit the utility of the HA degradation process as a bulk bone substitute, these problems can be overcome due to the dispersion of HA in a compliant hydrogel matrix, effectively restraining crack propagation [16]. Previous studies have shown that HA reinforces gelatin-based matrices while enhancing osteogenic activity [2,17,18]; for instance, incorporating hydroxyapatite (and strontium-doped HA) into a gelatin methacryloyl matrix improved the mechanical, swelling, and degradation behavior of the printed hydrogel while supporting osteoblast adhesion and proliferation [18].

Methylcellulose has previously been used to improve the printability of gelatin-based inks, while hydroxyapatite has been incorporated into gelatin- or GelMA-based scaffolds to promote osteogenesis, often using photocrosslinking [11,12,13,14]. However, the systematic integration of MC, gelatin, and HA within a single EDC/NHS-crosslinked, non-photocrosslinked ink, and the effects of MC content at a fixed HA loading on rheology, printing fidelity, mechanical performance, and osteogenic response, have not been systematically investigated.

Here, we developed an EDC/NHS-crosslinked MC/GEL/HA ink combining the cell-adhesive properties of gelatin, the rheological and printing functions of MC, and the osteoconductive properties of HA. MC content was systematically varied (10–15% *w*/*v*) across HA-containing formulations at a fixed HA loading, with corresponding HA-free formulations included as controls. We systematically assessed their rheological, mechanical, and biological performance using human bone marrow-derived mesenchymal stem cells (hBM-MSCs). We hypothesized that optimizing the formulation would improve printing fidelity while promoting osteogenic activity suitable for patient-specific repair of non-weight-bearing bone defects, including those of the craniofacial skeleton.

## 2. Materials and Methods

### 2.1. Hydrogel Preparation

**Figure 1:**
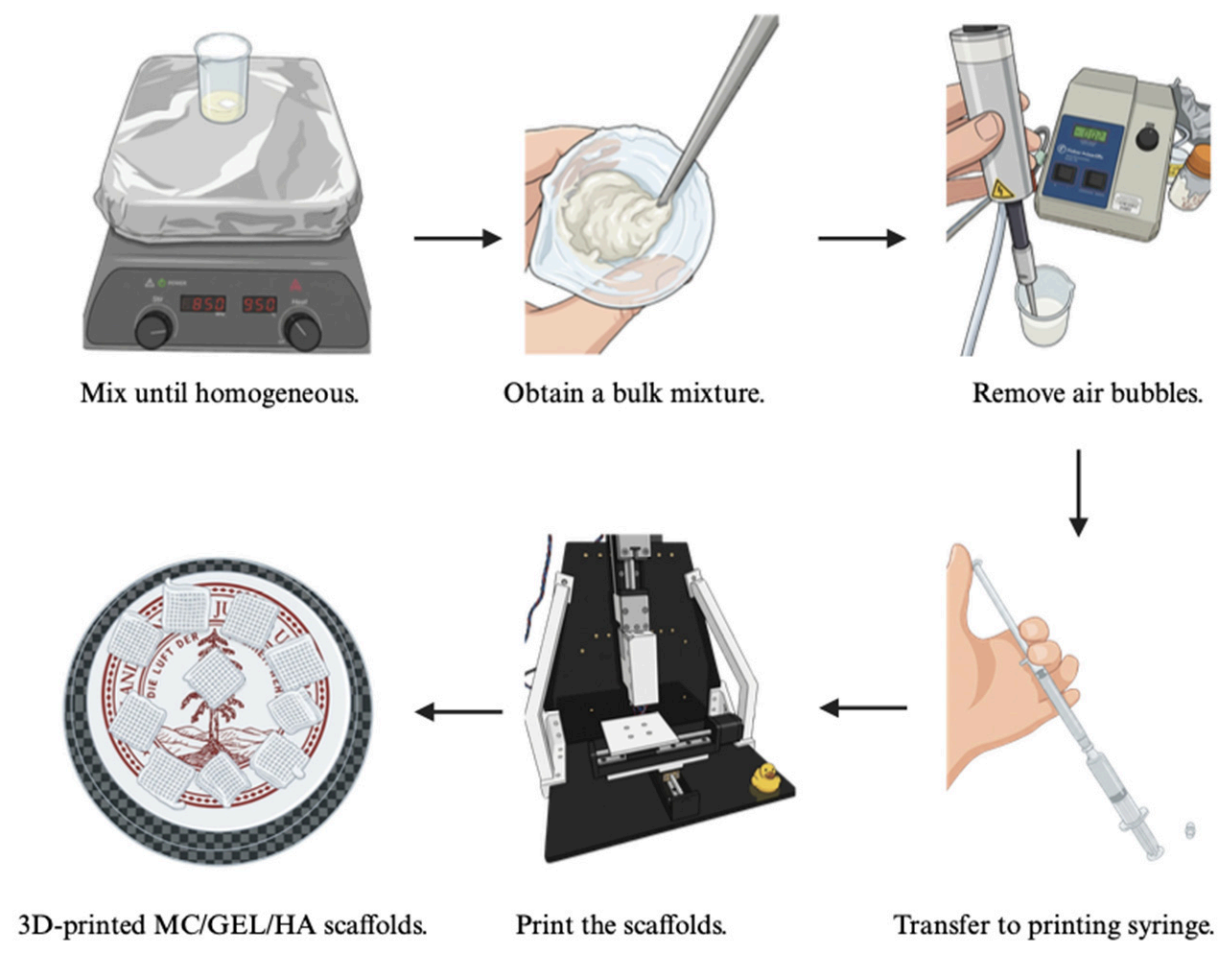
Protocol for preparation of ink. Created in BioRender. Yuksel, C. (2026) https://BioRender.com/skhchow (Accessed on 26 July 2026).

MC/GEL/HA was blended at various concentrations (Table 1). MC powder was dissolved in distilled water at 50 °C under continuous stirring and hydrated overnight at 4 °C [19]. Gelatin was separately dissolved in distilled water at 50 °C [20], followed by gradual addition of HA powder and incorporation of the MC solution to form the composite hydrogel. The mixture was sonicated to remove air bubbles, loaded into a printing syringe, which was held at 50 °C in a water bath before printing. The fabrication protocol (Figure 1) used an extrusion-based 3D printer according to predefined parameters.

### 2.2. 3D Printer Design and Scaffold Fabrication

Hydrogels were printed using the Printess: a custom-built, low-cost extrusion-based 3D printer developed at Stanford University, Stanford, CA, USA [21]. Scaffold geometry was defined by coordinate-based G-code to produce three-dimensional porous mesh structures. The hydrogel was maintained at 50 °C in a heated block and extruded through a 25 G needle [22]. Extrusion parameters were adjusted per formulation viscosity: E1.20 (E/mm = 0.08), E1.50 (E/mm = 0.10), and E1.80 (E/mm = 0.12) for 10%, 12%, and 15% MC, respectively, per 15 mm printing path. Following printing, scaffolds were crosslinked in 50 mM 1-Ethyl-3-(3-dimethylaminopropyl) carbodiimide/ N-Hydroxysuccinimide (EDC/NHS) at 4 °C for 20 h to form amide bonds between gelatin amino groups. After crosslinking, scaffolds were washed three times with phosphate-buffered saline (PBS) to remove residual reagents [22].

### 2.3. Rheological Analysis

Rheological measurements were acquired on an Anton Paar rotational rheometer (Anton Paar GmbH, Graz, Austria) using a 15 mm parallel-plate geometry and a 1 mm gap. The linear viscoelastic region (LVR) was determined by oscillatory amplitude sweeps at 37 °C and 10 rad s^−1^ over a strain range of 0.01–100%, from which the storage (G′) and loss (G″) moduli were obtained. Steady-shear measurements were then conducted over a shear rate range of 0.01–100 s^−1^ to evaluate the shear-thinning behavior. Thermal gelation behavior was characterized by temperature sweeps from 25 °C to 80 °C at a heating rate of 10 °C min^−1^, recording complex viscosity (η*) and G′/G″; the gelation temperature was taken as the G′-G″ crossover [23]. All rheological measurements were performed at Boğaziçi University (Istanbul, Türkiye).

### 2.4. Fourier-Transform Infrared Spectroscopy (FTIR)

FTIR spectroscopy was carried out to identify the functional groups and chemical interactions within the MC/GEL/HA hydrogels. Measurements were acquired over the 400–4000 cm^−1^ range on a Nicolet iS50 spectrometer (Thermo Fisher Scientific, Waltham, MA, USA).

### 2.5. Pore Size and Filament Diameter Analysis

Scaffold morphology, porous architecture, pore size, and filament diameter were examined by scanning electron microscopy (SEM) [24]. Prior to imaging, scaffolds were EDC/NHS crosslinked, frozen at −20 °C, and lyophilized, then mounted onto aluminum stubs with double-sided conductive carbon tape. Imaging was conducted on a FEI-Philips XL30 field-emission scanning electron microscope (Boğaziçi University, Istanbul, Türkiye) in low-vacuum mode at 20 kV, using a low-vacuum secondary-electron detector (LVD) [22]. Pore size and filament diameter were quantified from at least three randomly selected fields per scaffold using ImageJ 1.54 (National Institutes of Health, Bethesda, MD, USA) following scale-bar calibration, and results were expressed as mean ± standard deviation.

The printability index (Pr) was calculated from the perimeter (L) and area (A) of pores in the printed grid structures [25]:

(1) $$Pr=\frac{L^{2}}{16A}$$

An ideal square pore geometry yields Pr = 1, whereas Pr < 1 indicates a more circular pore shape from filament fusion or spreading, and Pr > 1 indicates an irregular, non-uniform pore geometry [25]. Pore perimeter and area were measured using the same ImageJ protocol described above, from at least four pores per scaffold across three independent samples.

To define the reference geometry for morphological analysis, the theoretical filament width and pore size specified by the printing design were calculated. The programmed filament width was 1.0 mm and was identical across formulations because the same G-code geometry was used. For the 15 × 15 mm lattice containing 11 filaments per direction and ten intervening pores, the theoretical pore width was calculated as (15 − 11 × 1.0)/10 = 0.4 mm (400 μm).

### 2.6. Swelling/Degradation Tests and In Vitro pH Stability

The swelling and degradation behavior of the scaffolds was studied using a separate set of scaffolds for each time point (*n* = 3 per group). Each scaffold was first dried in an incubator at 37 °C for 24 h, and its initial dry weight (W_0_) was recorded. Scaffolds were then immersed in PBS at 37 °C. On days 1, 3, 7, 14, and 18, the corresponding scaffolds were removed from PBS, gently blotted to remove surface water, and weighed to obtain the swollen mass (W_s_). These scaffolds were then dried at 37 °C for 24 h and reweighed (Wd) to determine the mass loss for degradation analysis [26]. Swelling and degradation were expressed as percentages according to [27]:

(2) $$\%Water\;Uptake=\frac{\left(W_{s}-Wd\right)}{Wo}$$

(3) $$\%Weight\;Loss=\frac{\left(Wo-Wd\right)}{Wo}$$

The pH of the 3D-printed scaffolds was monitored over 18 days. Scaffolds from every experimental group were immersed in PBS at 37 °C (*n* = 3 per group). Fresh PBS was added daily to compensate for evaporation and maintain a constant medium volume during incubation [28]. The pH of the surrounding medium was measured daily with an Accumet pH meter (Fisher Scientific, Waltham, MA, USA). All experiments were conducted at Stanford University (Stanford, CA, USA).

In a complementary experiment, water uptake and mass loss of the MC/GEL and MC/GEL/HA scaffolds were monitored for 49 days in the complete cell culture medium used for the biological assays (DMEM Alpha supplemented with 10% fetal bovine serum and 1% penicillin-streptomycin), providing a biochemical environment that more closely resembles cell culture conditions, including exposure to serum proteins and growth factors [29]. After recording the initial dry weight (W_0_), scaffolds were incubated in the complete medium at 37 °C, with fresh medium replaced periodically to maintain nutrient availability. At predetermined time points (days 3, 7, 14, 21, 28, 35, 42, and 49), scaffolds were removed, gently blotted to remove excess medium, weighed (W_s_), and then dried for 24 h to determine the final dry weight (Wd). Water uptake and mass loss were calculated using the same equations described above.

### 2.7. Mechanical Testing

The mechanical properties of the scaffolds, including compressive strength and elasticity, were evaluated [30]. Uniaxial compression testing was carried out on an Instron 5565 universal testing machine (Instron, Norwood, MA, USA) equipped with a 100 N load cell. Specimens measuring 15 × 15 × 2 mm were tested at room temperature (*n* = 5 per group). Samples were compressed between two parallel platens at a crosshead speed of 1 mm min^−1^ until failure, and load–displacement data were converted into stress–strain curves [31]. The compressive modulus was determined from the slope of the initial linear region of the stress–strain curve (between 5% and 10% strain). Compressive strength was taken as the maximum stress at the point of failure, and the corresponding strain at failure (%) was also recorded. All values are reported as mean ± standard deviation. All experiments were performed at Stanford University (Stanford, CA, USA).

### 2.8. Contact Angle Measurement

Static water contact angle measurements were performed using the sessile drop method on a KSV CAM 101 goniometer (KSV Instruments, Helsinki, Finland) at Boğaziçi University, Istanbul, Türkiye. A droplet of deionized water was carefully placed onto the surface of each sample, and images were captured after 1 s at room temperature to minimize the effect of droplet spreading. Measurements were conducted in three independent replicates for each sample group (*n* = 3), and the contact angle values were reported as the average. All analyses were performed on bulk scaffold samples.

### 2.9. Bioactivity Studies (Simulated Body Fluid Test)

The in vitro bioactivity of the scaffolds was evaluated based on their ability to induce apatite formation in simulated body fluid (SBF), prepared according to Kokubo and Takadama [32,33]. Rectangular specimens (15 × 15 × 2 mm) were immersed in 15 mL of SBF at 37 °C for up to 14 days (*n* = 3 per group per time point), with the SBF refreshed every 3 days to preserve ionic equilibrium. Samples were retrieved at day 7 and day 14 to monitor the onset and progression of apatite deposition. After immersion, samples were rinsed with distilled water and dried at 37 °C.

Apatite deposits on the scaffold surfaces were examined with a JEOL JSM-IT500HR environmental scanning electron microscope. Energy Dispersive X-ray Spectroscopy (EDS) elemental mapping was used to determine the spatial distribution of calcium (Ca) and phosphorus (P), and the Ca/P molar ratio was calculated to assess how closely it matched that of native hydroxyapatite [34]. Post-immersion samples at both time points were further characterized by FTIR to identify characteristic bands associated with bone-like apatite formation [35].

### 2.10. Human Bone Marrow Mesenchymal Stem Cell Culture

Human bone marrow mesenchymal stem cells (hBM-MSCs) were maintained in DMEM Alpha (Biowest) supplemented with 10% fetal bovine serum and 1% penicillin-streptomycin (Biowest) at 37 °C under a humidified 5% CO_2_ atmosphere. Prior to seeding, MC/GEL/HA scaffolds were sterilized by UV irradiation (45 min per side). To prevent non-specific cell attachment to the culture surface, the wells of 12-well plates were precoated with poly (2-hydroxyethyl methacrylate) (pHEMA), whereas TCP control wells were left uncoated [36]. hBM-MSCs were seeded directly onto the scaffolds at 1 × 10^6^ cells per well and allowed to attach for 2 h, after which 1 mL of complete medium was added and refreshed daily (*n* = 3 per group). Tissue culture plastic (TCP) seeded at the same density served as the positive control. All cell culture studies were performed at Stanford University (Stanford, CA, USA).

#### 2.10.1. Cell Viability and Proliferation

Cell metabolic activity was evaluated on days 3 and 7 using the Alamar Blue assay [37]. Alamar Blue (10% *v*/*v*) was added at each time point, and scaffolds were incubated for 4 h at 37 °C. Aliquots (100 μL) of the supernatant were transferred to a 96-well plate, and absorbance was recorded at 570 and 600 nm on a SpectraMax iD3 microplate reader [38]. Values were normalized to the day-3 TCP control (set to 100%) [39].

#### 2.10.2. DAPI/Phalloidin Staining

Cell attachment, morphology, and cytoskeletal organization were examined on days 3 and 7. Cell-seeded scaffolds were fixed with 4% paraformaldehyde for 15 min, permeabilized with 0.1% Triton X-100 for 10 min, and blocked with 1% bovine serum albumin for 30 min, with PBS washes between steps. F-actin was labeled with Phalloidin (30 min) [40] and nuclei counterstained with 4′,6-diamidino-2-phenylindole (DAPI) (20 min), with all steps from this point performed in the dark to limit photobleaching. Samples were imaged on a BZ-X810 fluorescence microscope and analyzed using BZ-X800 Viewer software (version 1.3.0.0, KEYENCE, Osaka, Japan).

#### 2.10.3. Alkaline Phosphatase (ALP) Staining

Early osteogenic commitment was assessed by alkaline phosphatase staining after 7 days of culture in osteogenic medium (DMEM supplemented with 10% FBS, 1% penicillin-streptomycin, 100 μg mL^−1^ ascorbic acid, 10 mM β-glycerophosphate, and 100 nM dexamethasone). hBM-MSCs were seeded at 1 × 10^6^ cells per well (*n* = 3 per group), with acellular scaffolds processed identically serving as the background control [41]. Samples were rinsed with PBS, fixed with 4% paraformaldehyde for 15 min, and rinsed again with PBS. ALP activity was visualized using NBT/BCIP substrate (Thermo Fisher Scientific, Waltham, MA, USA), and samples were incubated at 37 °C until color development, after which the reaction was stopped by rinsing with distilled water [42]. Stained samples were imaged in bright-field mode on a BZ-X810 microscope (Keyence, Osaka, Japan). Stained area was measured from three independent samples per group using ImageJ (NIH, USA) [43].

#### 2.10.4. Alizarin Red S Staining

Calcium deposition was evaluated by Alizarin Red S staining after 14 days of culture in osteogenic medium [44]. hBM-MSCs were seeded at 1 × 10^6^ cells per well in 12-well plates (*n* = 5 per group) and fixed with 4% paraformaldehyde for 15 min. To confirm the presence of cells within the mineralized regions, nuclei were first stained with DAPI (1 μg/mL, 10 min) and imaged under fluorescence microscopy. The same samples were then stained with 2% (*w*/*v*) Alizarin Red S (pH 4.2) for 30 min, after which excess dye was removed with distilled water. Mineralized nodules were imaged on a BZ-X810 fluorescence microscope (Keyence, Osaka, Japan) and analyzed with BZ-X800 Viewer software, with red-stained deposits taken as evidence of matrix mineralization. For quantification, bound stain was extracted in 20% (*v*/*v*) methanol/10% (*v*/*v*) acetic acid, and absorbance was measured at 450 nm [44]; acellular scaffolds processed identically were stained and quantified in parallel, and their absorbance values were subtracted as background.

#### 2.10.5. Reverse Transcription Quantitative PCR (RT-qPCR)

Osteogenic gene expression was evaluated in the hydroxyapatite-containing groups. Osteocalcin (OCN), a late-stage marker of mature osteoblast activity and bone matrix formation, was quantified on days 7 and 14 of osteogenic culture [45]. Expression was normalized to GAPDH using the ΔCt method [46]. Primer sequences are listed in Table 2. All reactions were run in triplicate.

##### RNA Extraction

Total RNA was isolated using TRIzol under RNase-free conditions. TRIzol reagent (Thermo Fisher Scientific, Waltham, MA, USA) was added directly to each well containing the cell-seeded scaffolds (*n* = 3 per group), and the lysate was collected by pipetting. Chloroform was added for phase separation, and samples were centrifuged at 12,000× *g* for 15 min at 4 °C. RNA in the aqueous phase was precipitated with isopropanol using glycogen as a carrier, washed with 75% ethanol, air-dried, and resuspended in RNase-free water [28,46]. RNA concentration and purity were assessed on a NanoDrop One spectrophotometer (Thermo Fisher Scientific, Waltham, MA, USA), with A260/A280 ratios of 1.8–2.0 considered acceptable.

##### Complementary DNA (cDNA) Synthesis

cDNA was synthesized from total RNA using iScript Reverse Transcription Supermix (Bio-Rad) in 20 μL reactions assembled on ice. Reverse transcription was performed in a MiniAmp Plus thermal cycler (Thermo Fisher Scientific, Waltham, MA, USA) using the following thermal profile: priming at 25 °C for 5 min, reverse transcription at 46 °C for 20 min, and reverse transcriptase inactivation at 95 °C for 1 min. Products were stored at −20 °C.

##### qPCR

cDNA was diluted 1:5 in RNase-free water and amplified using PowerUp SYBR Green Master Mix (Applied Biosystems, Waltham, MA, USA) with primers for OCN and GAPDH (Table 2), on a QuantStudio 6 Pro Real-Time PCR System (Applied Biosystems, Waltham, MA, USA); specificity was confirmed by melt-curve analysis. Relative OCN expression was expressed as 2^−ΔCt^. Data were processed in Design and Analysis Software v2.6.0 (Applied Biosystems).

### 2.11. Statistical Analyses

Statistical analysis was performed using GraphPad Prism (version 11.0.2; GraphPad Software, Boston, MA, USA). Group differences were assessed by one-way ANOVA with Tukey’s post hoc test, except for cell viability (7 × 2; group × time point), swelling and degradation (6 × 5; group × time point), pH (6 × 18; group × time point), and OCN expression (3 × 2; group × time point) data, which were analyzed by two-way ANOVA with Tukey’s post hoc test, with statistical significance set at *p* < 0.05 [47]. Effect size was calculated as eta-squared (η^2^) for one-way ANOVA and partial eta-squared (partial η^2^) for two-way ANOVA comparisons to determine the magnitude of observed differences [48,49].

## 3. Results and Discussion

### 3.1. Rheological Characterization of Hydrogel Formulations

**Figure 2:**
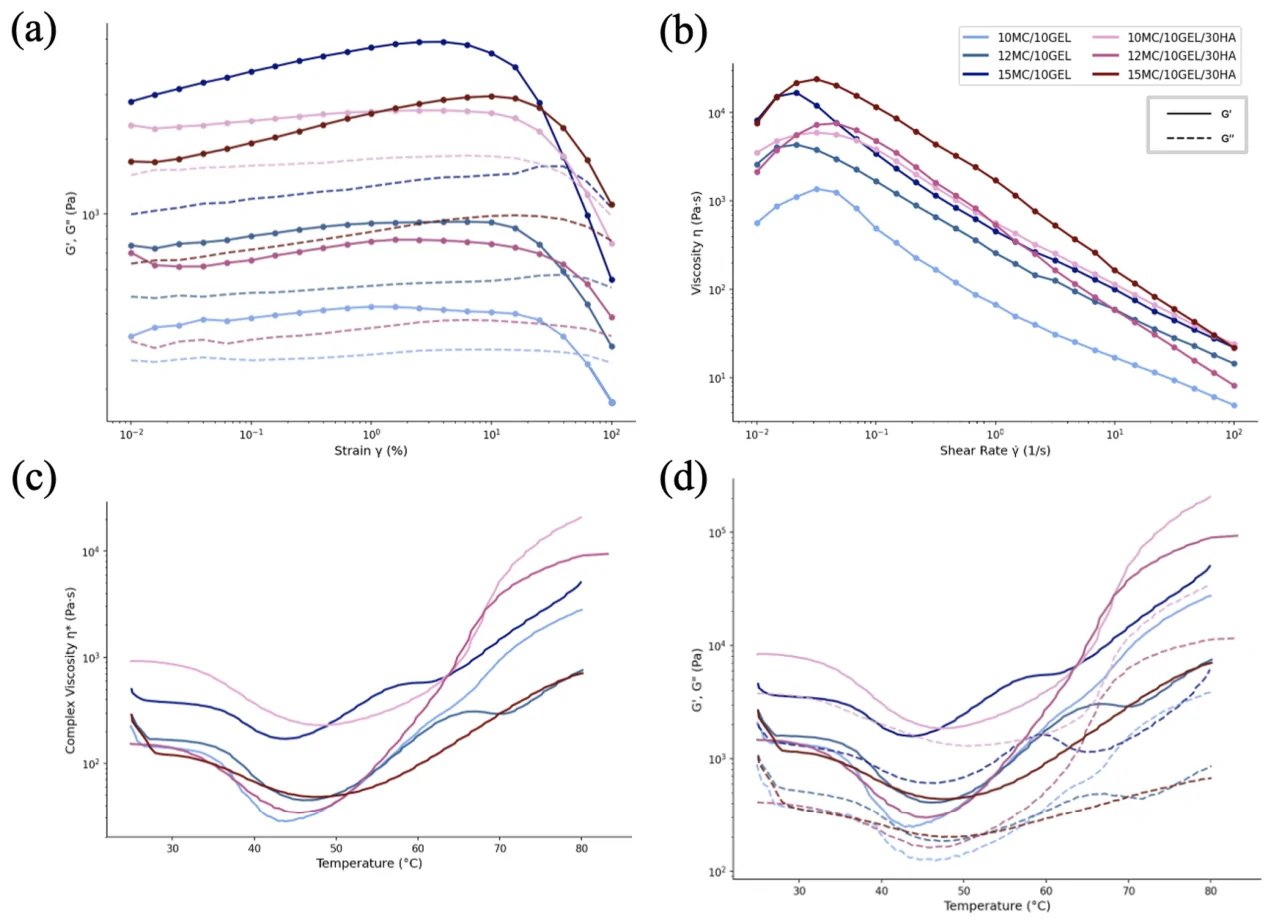
Rheological properties of the MC/GEL/HA hydrogels evaluated through: (**a**) amplitude sweep, (**b**) shear rate- dependent viscosity, (**c**) complex viscosity variations with temperature, and (**d**) temperature sweep analysis.

The rheological properties of the MC/GEL/HA hydrogels were evaluated by amplitude sweep, shear-rate-dependent viscosity, temperature-dependent complex viscosity, and temperature-dependent modulus measurements (Figure 2a–d).

The amplitude sweep measurements (Figure 2a) revealed composition-dependent differences in the viscoelastic behavior of the MC/GEL/HA hydrogels. Across all formulations, G′ exceeded G″ throughout the linear viscoelastic region before both moduli declined sharply at higher % strains, indicating predominantly elastic gel behavior followed by network disruption [50]. MC concentration was the primary determinant of gel stiffness, with G′ increasing progressively from 10MC/10GEL (the lowest modulus) to 15MC/10GEL [12]. The influence of HA depended on MC concentration, substantially increasing G′ in the 10MC/10GEL formulation but producing only minor changes in the 12MC/10GEL and 15MC/10GEL formulations [51,52].

Shear-rate-dependent viscosity (Figure 2b) characterized the flow behavior of the hydrogels across a shear-rate range of 0.01–100 s^−1^. All six formulations displayed pronounced shear-thinning, with viscosity decreasing by two to three orders of magnitude as the shear rate increased, a characteristic essential for extrusion-based printing [53,54]. The elevated viscosity at low shear rates (≤0.1 s^−1^), prior to the onset of shear-thinning, is indicative of yield-stress-like behavior. Viscosity also increased with MC concentration, confirming that higher MC content strengthened the network under flow. The HA-containing formulations, particularly 15MC/10GEL/30HA, showed the highest viscosities, suggesting that HA further contributed to flow resistance under shear.

Temperature-dependent complex viscosity (Figure 2c) revealed the temperature behavior of the hydrogels. All formulations displayed a reduction in complex viscosity upon initial heating, reaching a minimum between approximately 44 and 49 °C, followed by a rapid increase with further heating. This behavior reflects the coil-to-fibril transition characteristic of MC gels, in which polymer chains reorganize from disordered coils into fibrillar aggregates above a critical temperature, producing macroscopic stiffening [55,56,57,58]. The similar transition temperatures indicate that all formulations underwent the same thermal gelation mechanism, with only the magnitude of the response varying among compositions. Based on these results, 50 °C was chosen as the processing temperature for subsequent experiments, since it provided an intermediate viscosity following the onset of thermal gelation while avoiding the substantially higher viscosities observed at 70–80 °C.

Figure 2d presented the corresponding temperature sweep for G′ and G″. In contrast to hydrogel systems that display a sol–gel crossover upon heating [59], G′ remained above G″ across the entire 25–80 °C range for every formulation, confirming that gel-like character was preserved throughout the tested temperature range. Both moduli decreased until approximately 45–50 °C before increasing again, mirroring the trend observed in the complex viscosity data. Among the HA-free formulations, thermal stiffening became more pronounced with increasing MC content, from 10MC/10GEL to 12MC/10GEL and 15MC/10GEL. The contribution of HA to thermal stiffening was greatest at low MC content, consistent with the pattern observed in the amplitude sweep [60].

MC concentration emerged as the dominant factor governing the viscoelastic behavior of the MC/GEL/HA system, while HA acted as a composition-dependent reinforcing phase most effective in low-MC networks [51,52,60]. All formulations nonetheless showed gel-like behavior, pronounced shear-thinning, and thermally induced stiffening, supporting their suitability as inks for extrusion-based 3D printing.

### 3.2. FTIR Analysis and Surface Morphology of Scaffolds

**Figure 3:**
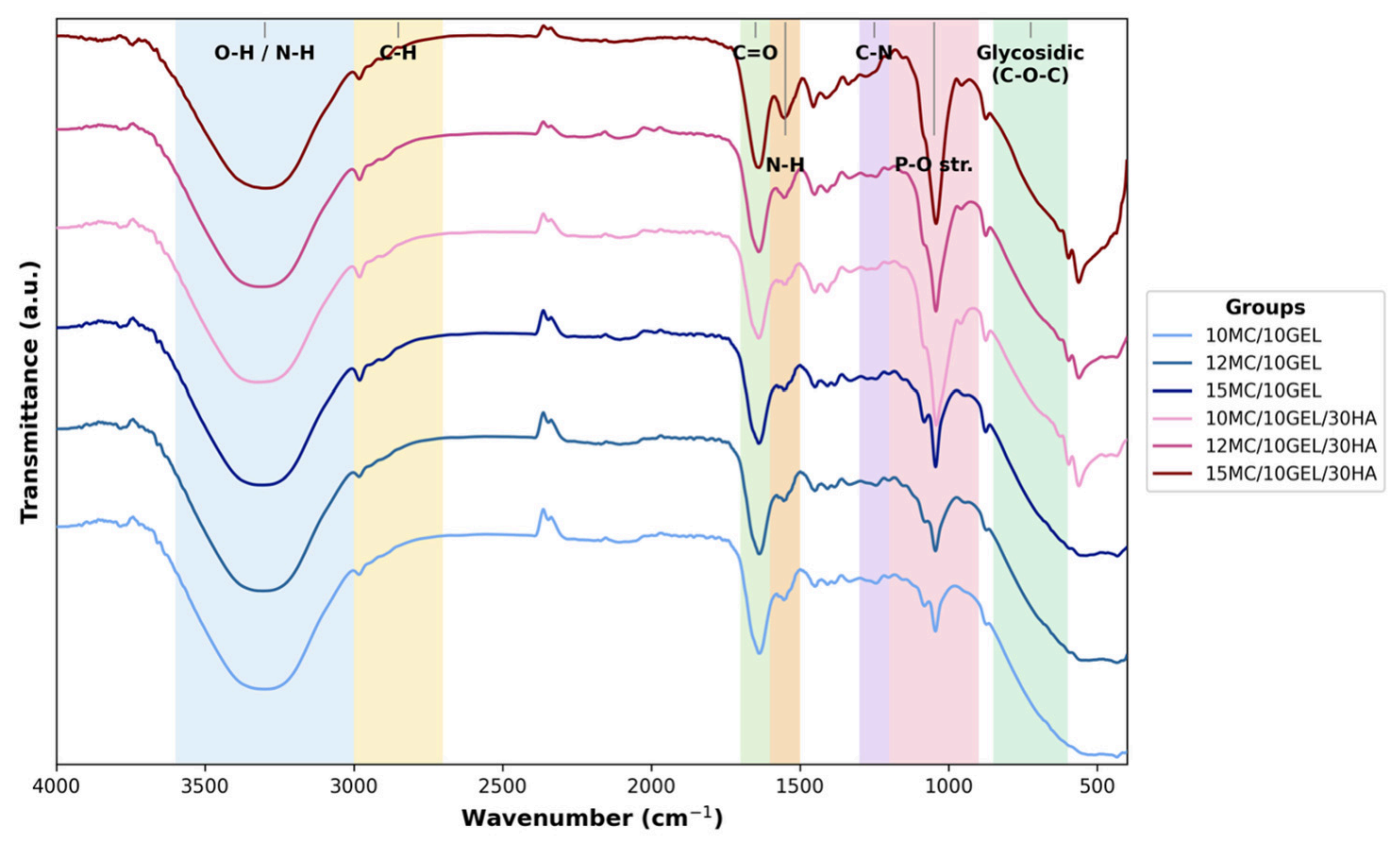
FTIR-ATR spectroscopy of EDC/NHS cross-linked MC/GEL and MC/GEL/HA scaffolds (vertically shifted for visibility). Shaded regions mark diagnostic bands: O-H/N-H stretching (3600–3000 cm^−1^), C-H stretching (~2900 cm^−1^), amide I (~1650 cm^−1^), amide II (~1550 cm^−1^), C-N stretching (~1250 cm^−1^), P-O stretching (1100–950 cm^−1^), and O-P-O bending (~600 cm^−1^).

FTIR spectra of all EDC/NHS-crosslinked formulations (Figure 3) retained the characteristic bands of MC and gelatin, including broad O-H/N-H stretching (3000–3600 cm^−1^), C-H stretching (~2900 cm^−1^), and the amide I and II bands (~1630–1650 and ~1540–1560 cm^−1^; corresponding to C=O stretching and combined N-H bending/C-N stretching, respectively), indicating preservation of the chemical spectral features of the MC/GEL matrix across all formulations [61].

The prominent amide I and II bands, together with the absence of a distinct free-carboxyl shoulder (~1710–1740 cm^−1^), suggest amide bond formation through EDC/NHS-mediated coupling, in agreement with previous reports describing carbodiimide crosslinking of gelatin-based systems [59,62].

HA-containing formulations displayed additional phosphate-associated absorptions, including P-O stretching (900–1100 cm^−1^) and O-P-O bending (~500–650 cm^−1^), characteristic of calcium phosphate phases [63]. Although the 900–1100 cm^−1^ region partially overlaps with C-O-C vibrations of the MC/GEL matrix, the increased intensity of these phosphate-related bands relative to HA-free controls is in line with the presence of HA.

These spectral features confirm that HA was successfully incorporated into the EDC/NHS-crosslinked MC/gelatin matrix.

### 3.3. 3D Printing of Scaffolds

**Figure 4:**
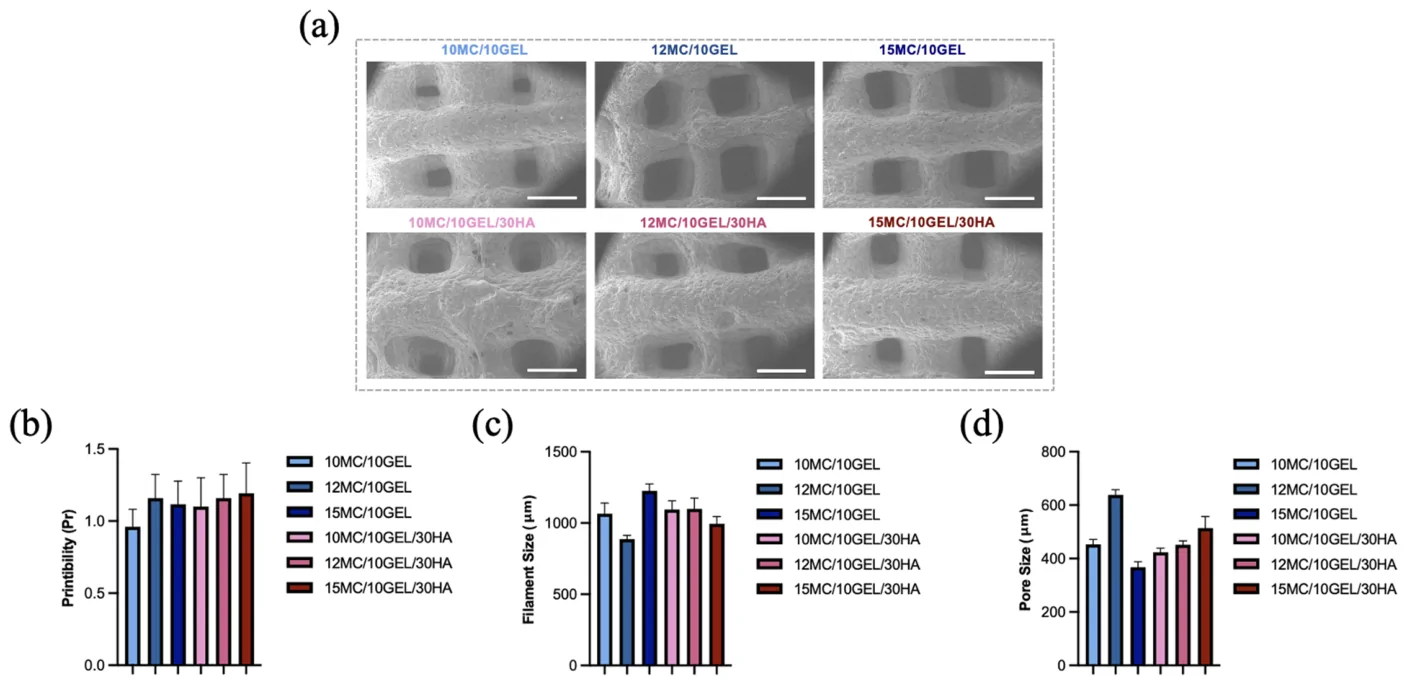
Morphological and structural characterization of MC/GEL and MC/GEL/HA scaffolds. (**a**) Representative SEM images of printed scaffolds showing the interconnected, square-pore architecture of 10MC/10GEL, 12MC/10GEL, and 15MC/10GEL formulations (top row) and their HA-containing counterparts, 10MC/10GEL/30HA, 12MC/10GEL/30HA, and 15MC/10GEL/30HA (bottom row). Scale bars, 50 μm. (**b**) Printability index (Pr), calculated from the perimeter and area of printed pores, with Pr = 1 indicating an ideal square pore geometry. (**c**) Filament diameter and (**d**) Pore size measured from SEM images. Data are presented as mean ± standard deviation (*n* = 3 per group); differences not indicated are non-significant.

SEM imaging demonstrated that all scaffold groups maintained a well-defined, interconnected square-pore structure following printing (Figure 4a). The 0°/90° strand deposition pattern remained intact, with no visible strand collapse or fusion observed in any group, indicating good shape fidelity across all compositions. Quantitative printability (Pr) analysis supported these SEM findings, with Pr values clustering near 1 across all groups (Figure 4b), indicating near-ideal pore geometry. Groups containing HA showed a slight increase in Pr, consistent with decreased strand spreading after extrusion.

Filament diameter (Figure 4c) ranged from roughly 850 to 1200 μm, with the highest value observed in the 15MC/10GEL group. This pattern is likely attributable to the greater viscosity of MC-rich inks (described in Figure 2b), which produces thicker extruded filaments. The effect of HA on filament diameter depended on MC content: HA increased filament diameter at the two lower MC concentrations (10MC/10GEL, 12MC/10GEL) but reduced it at the highest MC concentration (15MC/10GEL), suggesting reduced post-extrusion filament expansion at higher MC content.

The pore sizes (Figure 4d) ranged from approximately 350 to 610 μm, falling within the range generally considered favorable for bone regeneration, where interconnected pores support cell infiltration, nutrient transport, and vascularization [64]. Even the smallest pores exceeded the lower thresholds commonly associated with restricted cell migration. Accordingly, MC and HA provided complementary control over filament and pore dimensions without compromising print fidelity.

Relative to the theoretical design values (Section 2.5; 1.0 mm programmed filament width and 400 μm theoretical pore width), the SEM-measured filament diameters ranged from approximately 850 to 1200 μm, whereas pore sizes ranged from approximately 350 to 610 μm. Thus, the printed filaments generally exceeded the programmed width, with a corresponding reduction in pore size. The magnitude of this deviation varied with formulation and was most pronounced at higher MC content, consistent with the greater viscosity and flow resistance of these inks (Figure 4c,d).

This trend is consistent with the rheological behavior described in Section 3.1. HA incorporation increased viscosity and low-shear resistance to flow (Figure 2b), particularly in the lower-MC formulations, which can limit strand spreading and relaxation after deposition. Reduced spreading preserves more defined strand boundaries and alters pore geometry, contributing to the observed changes in pore size and printability index. Because pore size is constrained by the fixed strand spacing of the printed lattice and the deposited filament width, formulation-dependent changes in filament dimensions directly translate into corresponding changes in pore size.

### 3.4. Compressive Behavior of the Scaffolds

**Figure 5:**
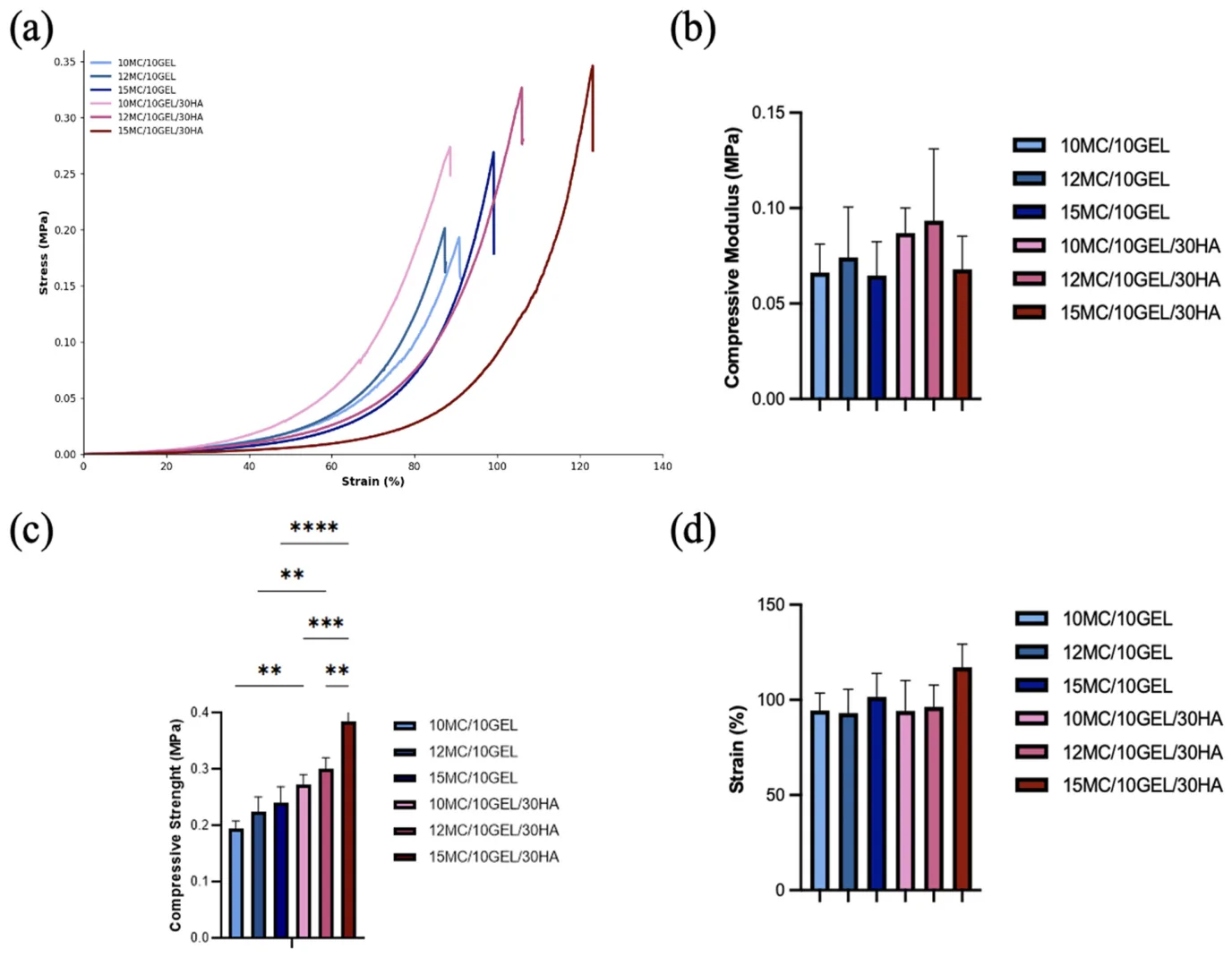
Compressive mechanical behavior of MC/GEL and MC/GEL/HA scaffolds. (**a**) Representative uniaxial compressive stress–strain curves, (**b**) compressive modulus, (**c**) compressive strength, and (**d**) % strain, derived from the curves in (**a**). Data are presented as mean ± s.d. (*n* = 5). Statistical significance was assessed by one-way ANOVA with Tukey’s post hoc test (** *p* < 0.01, *** *p* < 0.001, **** *p* < 0.0001; differences not indicated are non-significant).

The mechanical properties of the MC/GEL-based hydrogels were evaluated under uniaxial compression (Figure 5). Stress–strain curves (Figure 5a) exhibited the characteristic J-shaped behavior, with an initially compliant response followed by progressive strain stiffening, typical of hydrated polymer networks [65,66].

The compressive modulus of the MC/GEL-based scaffolds is shown in Figure 5b. Neither increasing MC content nor incorporating HA produced a statistically significant change in compressive modulus, with values clustering between ~0.065 and ~0.093 MPa across all six groups. As the compressive modulus was determined within the low-strain compliant region governed by the soft, water-swollen polymer network, HA appears to contribute minimally to the initial mechanical response, with its reinforcing effect becoming more evident at larger compressive deformations [65,66].

Figure 5c shows the ultimate compressive strength of the MC/GEL-based scaffolds. Ultimate compressive strength increased progressively with increasing MC content in both the HA-free and HA-containing groups. Mechanistically, the MC-driven increase in compressive strength is consistent with the higher storage modulus and shear viscosity observed with increasing MC content (Section 3.1), suggesting a more physically interconnected polymer network with greater resistance to deformation.

Within the HA-containing groups, ultimate compressive strength increased from approximately 0.27 MPa (10MC/10GEL/30HA) to approximately 0.38 MPa (15MC/10GEL/30HA), with the greatest strength observed for 15MC/10GEL/30HA (*p* < 0.001 versus 10MC/10GEL/30HA). Compared with its HA-free counterpart, the 15MC/10GEL/30HA scaffold also exhibited higher ultimate compressive strength (*p* < 0.0001), suggesting additional reinforcement through interactions between HA particles and the surrounding polymer network, allowing the rigid ceramic phase to contribute more effectively to compressive load bearing in non-weight-bearing applications [67,68]. Additional non-covalent interactions, including hydrogen bonding between HA surface groups and the polymer network, may further contribute to interfacial load transfer. However, these interactions were not directly characterized here and would require dedicated interfacial studies to establish their contribution. Together, these findings indicate that MC and HA enhance compressive strength through complementary mechanisms.

In Figure 5d, the % strain at ultimate compressive strength showed little variation with increasing MC content, indicating that MC addition alone did not substantially alter network deformability. Although 15MC/10GEL/30HA showed a numerically higher % strain (~117%) compared with 15MC/10GEL (~101%), this difference did not reach statistical significance, and no significant differences in % strain were observed across any of the six formulations. Accordingly, neither MC content nor HA incorporation substantially altered scaffold deformability at the point of ultimate compressive strength.

### 3.5. Swelling, Degradation and pH Behavior of the Scaffolds

Swelling, weight loss (%) and pH were monitored in PBS at 37 °C over an 18 day degradation period (*n* = 3 per group), to evaluate scaffold stability under physiological conditions and the effect of HA incorporation.

**Figure 6:**
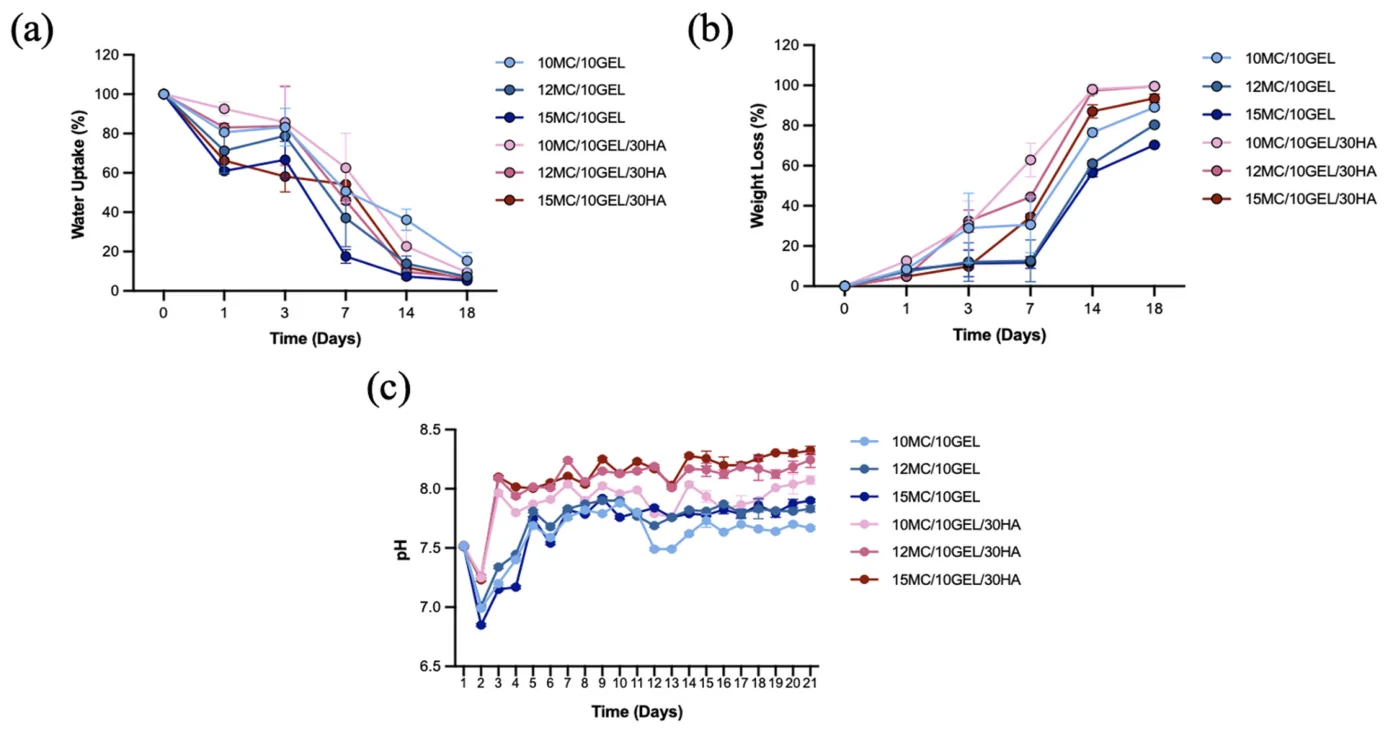
Physicochemical properties of MC/GEL and MC/GEL/HA scaffolds after treatment with PBS at 37 °C. (**a**) Percent water uptake over 18 days. (**b**) Percent weight loss over 18 days. (**c**) pH measurements of the 3D-printed scaffolds over 18 days. Data are presented as mean ± standard deviation (*n* = 3).

Water uptake (%) (Figure 6a) declined progressively over the immersion period rather than exhibiting classical swelling behavior: following a moderate initial decrease by day 1, values partially plateaued (or slightly recovered in some HA-containing groups) through day 3, after which all groups underwent a pronounced decline between days 3 and 14, before stabilizing at low residual levels (<20%) through day 18. HA-containing scaffolds retained higher water uptake than their HA-free counterparts for longer, with the greatest difference observed at day 7, before both groups converged to comparably low values by day 14.

Weight loss (%) (Figure 6b) followed the opposite trend. Scaffolds retained the majority of their initial mass during the first week, followed by a marked decline after day 7, with HA-containing groups degrading somewhat more rapidly than HA-free groups, consistent with their water uptake, which facilitated enhanced fluid penetration and promoted hydrolytic degradation of the polymer network [69,70]. By day 18, all groups had converged toward a low residual mass, indicating progressive degradation. Such gradual degradation is generally regarded as advantageous for bone tissue engineering, as it may provide transient structural support while allowing progressive tissue replacement [71].

Solution pH (Figure 6c) exhibited an initial decrease followed by recovery and stabilization. All groups revealed mild acidification of the environment in the first two days (minimum pH ≈ 6.9), which can be related to hydration of the matrix and release of gelatin’s residual species into the medium [72]. Starting from day 3, pH increased gradually before stabilizing by approximately day 7. HA-containing scaffolds equilibrated at distinctly alkaline pH values (approximately pH = 8.0–8.3), whereas HA-free scaffolds remained closer to neutral pH, consistent with the buffering and ion-exchange behavior of HA [73,74]. Among HA-containing materials, 15MC/10GEL/30HA maintained the highest pH throughout, suggesting the strongest buffering capacity.

HA incorporation accelerated degradation while sustaining a mildly alkaline microenvironment, a physicochemical profile that may support osteogenesis during tissue regeneration [75,76].

These trends indicate an inverse temporal relationship between water uptake and mass loss: formulations and time windows associated with greater water retention (Figure 6a) preceded and overlapped with periods of more rapid mass loss (Figure 6b), most clearly reflected by the decline in water uptake between days 3 and 14 and the onset of marked mass loss after day 7. This pattern is consistent with a water-mediated degradation process, in which greater fluid uptake may facilitate polymer-network chain scission, such that formulations retaining more water for longer, notably the HA-containing scaffolds, subsequently exhibit earlier and somewhat faster mass loss than their HA-free counterparts.

### 3.6. Surface Wettability

**Figure 7:**
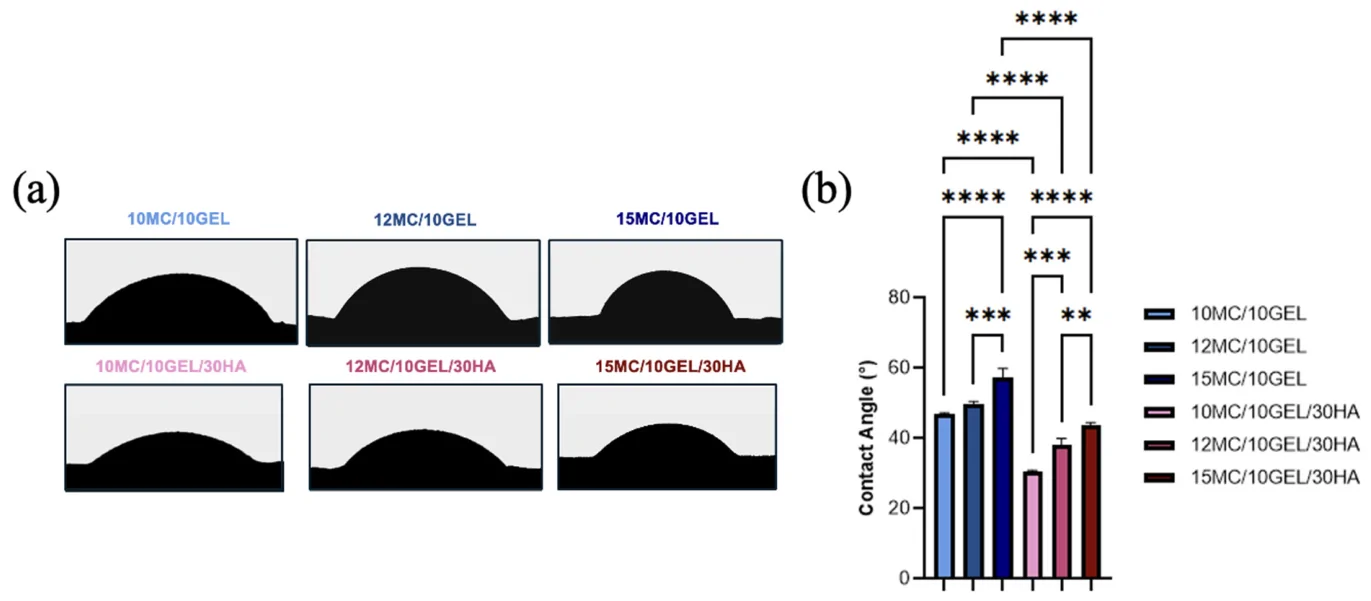
Surface wettability of the hydrogel formulations assessed by water contact angle measurement. (**a**) Representative sessile-drop images for the six formulations (10MC/10GEL, 12MC/10GEL, 15MC/10GEL, and their %30wt HA-containing counterparts). (**b**) Corresponding mean contact angles. All formulations exhibited hydrophilic surfaces (contact angle < 90°). Data are presented as mean ± SD (*n* = 3); asterisks denote statistical significance (** *p* < 0.01, *** *p* < 0.001, **** *p* < 0.0001); differences not indicated are non-significant.

Scaffold wettability was assessed through static water contact angle measurements, recorded 1 s after droplet deposition (Figure 7b), with representative sessile drop profiles presented in Figure 7a. All six formulations exhibited contact angles well below 90°, indicating hydrophilic surfaces [77]. In the series without HA, increasing the MC fraction resulted in progressively higher contact angles, from 46.2 ± 1.3° for 10MC/10GEL to 49.7 ± 1.1° for 12MC/10GEL and 57.1 ± 3.4° for 15MC/10GEL, with all pairwise differences being statistically significant (Figure 7b). The progressive increase in contact angle with MC content suggests enhanced hydrophobic association among methoxy-substituted chains, thereby reducing surface wettability, consistent with previous observations [78]. Incorporation of 30 wt % HA consistently decreased the contact angle across all formulations, to 30.4 ± 1.5°, 37.8 ± 2.2°, and 43.6 ± 1.4° for the 10, 12, and 15MC variants, respectively, while preserving the MC-dependent trend. This indicates that HA enhanced surface hydrophilicity while maintaining the compositional influence of the polymer network, a behavior commonly ascribed to the abundance of surface hydroxyl groups on HA, which readily engage in hydrogen bonding with water molecules [79].

### 3.7. Surface Morphology and Chemical Analysis of Apatite Formation

**Figure 8:**
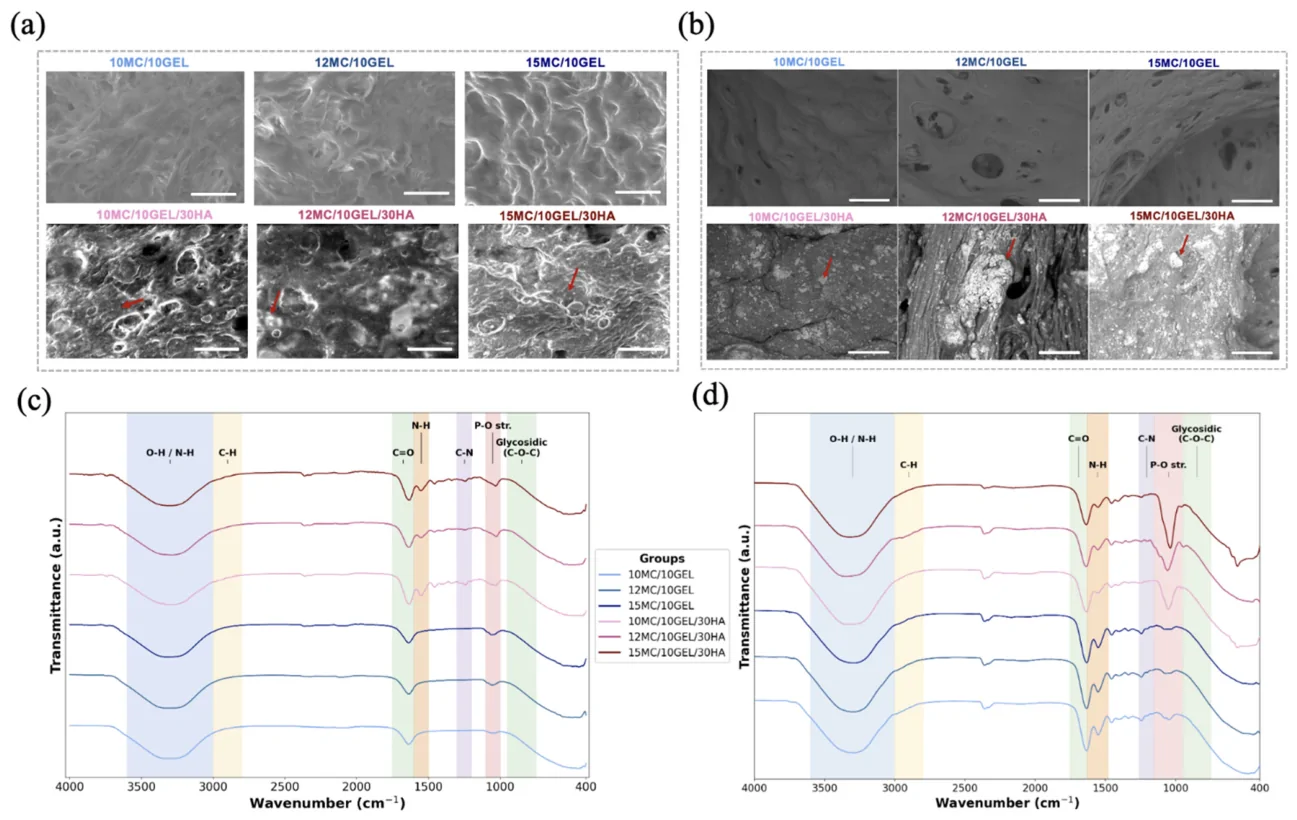
Apatite formation on the scaffolds after immersion in simulated body fluid. (**a**) SEM images and corresponding Ca/P elemental maps of all groups at day 7, showing Ca and P signals co-localized with surface deposits, with intensity increasing with MC content. Scale bars, 50 µm. (**b**) SEM images of the scaffold surface at day 14 in all groups (arrows mark apatite deposition). Scale bars, 50 µm. (**c**) FTIR spectra of the scaffolds at day 7, demonstrating phosphate and carbonate bands of the carbonated apatite phase. (**d**) FTIR spectra of the scaffolds at day 14, revealing restoration and enhancement of the P-O stretching band in the presence of HA.

Apatite-forming ability was evaluated after SBF immersion for up to 14 days using complementary SEM, EDS and FTIR analyses (Figure 8) to probe in vitro bioactivity, a widely used method for assessing in vivo bone-bonding potential [33].

SEM revealed particulate surface deposits (arrows, Figure 8a, bottom row), indicative of apatite nucleation. By day 7, isolated nodules were already present in the HA-containing formulations, while HA-free formulations showed no discernible particulate deposits at this time point (Figure 8a, top row). By day 14, the HA-containing groups displayed a denser, more continuous apatite layer (Figure 8b, bottom row), whereas the HA-free groups exhibited only a smooth, fibrous surface morphology without evidence of apatite deposition (Figure 8b, top row).

EDS was employed to quantify surface Ca/P atomic ratios, providing elemental confirmation that the SEM-observed deposits corresponded to an apatite-like calcium phosphate phase. At day 7, Ca/P ratios for 10MC/10GEL/30HA, 12MC/10GEL/30HA, and 15MC/10GEL/30HA were 1.83, 1.84, and 1.99, respectively, exceeding the 1.67 value expected for stoichiometric hydroxyapatite [80], consistent with the onset of apatite mineralization. By day 14, Ca/P ratios remained at similar values (1.85, 1.87, and 1.96), reflecting continued apatite formation. HA-free groups remained near the detection limit at both time points (0.33, 0.00, and 0.00 at day 7; 0.00–0.01 at day 14), indicating that the polymer matrix alone did not support heterogeneous apatite nucleation within this window.

Although phosphate-associated absorptions, together with weaker carbonate bands, were detectable from day 7 (Figure 8c), the intensity of the P-O stretching region (950–1150 cm^−1^) did not increase monotonically with immersion time. Instead, it was transiently attenuated alongside the N-H, C=O, and glycosidic bands during early immersion, before recovering by day 14 (Figure 8d), particularly in the HA-containing groups. This behavior likely reflects two concurrent effects. First, ATR-FTIR probes only the outermost ~0.5–2 µm of the surface [81], so a thin early apatite layer adds little signal against the strong intrinsic P-O background from the 30 wt % HA already present in the scaffold. Second, early hydrolytic degradation of the gelatin/MC matrix likely released soluble, non-crosslinked chains, explaining the concurrent decline in matrix bands. By day 14, as degradation progressed, recovery of the P-O signal was consistent with continued maturation of the apatite layer.

### 3.8. In Vitro Cytocompatibility

**Figure 9:**
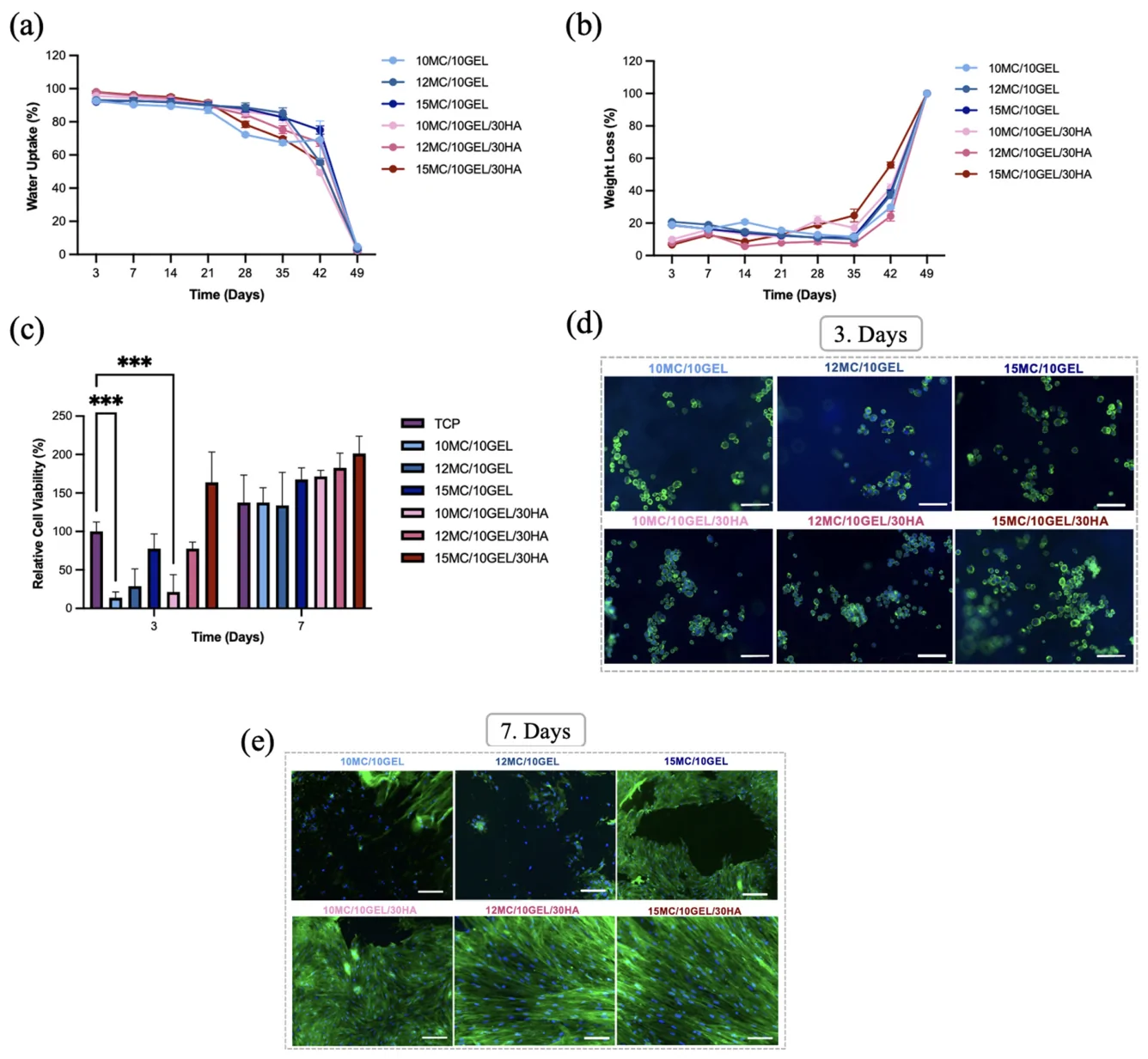
In vitro cytocompatibility and cell behavior of the hydrogel scaffolds. (**a**) Water uptake (%) of the six scaffold formulations over 49 days (days 3, 7, 14, 21, 28, 35, 42, 49) in cell culture medium. (**b**) Weight loss (%) of the same formulations over the same time course in cell culture medium. (**c**) Relative cell viability (%) of TCP (control) and the six formulations at days 3 and 7. (**d**) Representative fluorescence micrographs (DAPI/phalloidin; nuclei, blue; F-actin, green) of the six formulations at day 3. Scale bar, 100 μm. (**e**) Representative fluorescence micrographs of the same formulations at day 7. Scale bar, 100 μm. Data are mean ± s.d. (*n* = 3 per group); *** *p* < 0.001 (two-way ANOVA with Tukey’s post hoc test); differences not indicated are non-significant.

Water uptake (%) and mass loss (%) of the MC/GEL and MC/GEL/HA scaffolds were monitored over 49 days in the complete cell culture medium used for the biological assays (DMEM Alpha supplemented with 10% fetal bovine serum and 1% penicillin-streptomycin) (Figure 9a,b). This approach more closely reflects the biochemical environment experienced by cells during culture, including exposure to serum proteins and growth factors, thereby offering a more simulated physiologically relevant context for interpreting cell behavior on the scaffolds [30]. Water uptake (%) remained high (~90–95%) and relatively stable through day 21 for all formulations, then declined gradually between days 28 and 42, before dropping sharply for all groups by day 49. Weight loss (%) remained low and comparatively stable through day 21, then increased markedly after day 28, most notably for the higher-MC, HA-containing scaffold, before all groups converged toward near-complete mass loss by day 49.

The cytocompatibility of hBM-MSCs cultured on the MC/GEL and MC/GEL/HA scaffolds was assessed against a tissue-culture plastic (TCP) reference at days 3 and 7 (Figure 9c). At the earlier time point, most composite formulations supported a lower relative viability than TCP, most markedly for the 10MC/10GEL and 10MC/10GEL/30HA groups (both *p* < 0.001 vs. TCP). Increasing the MC content progressively improved day-3 viability, with 15MC/10GEL/30HA producing the highest response, although this did not differ significantly from TCP. This graded, dose-dependent effect of MC was also evident among the composite formulations, independent of the TCP comparison (e.g., 10MC/10GEL vs. 15MC/10GEL/30HA). By day 7, every composite condition had not only recovered but exceeded the TCP reference, with the HA-containing scaffolds showing the highest viability, and no statistically significant differences detected among the groups at this time point.

The composition-dependent response at day 3 is unlikely to be governed by wettability alone, since contact angle increased with MC content while day-3 viability increased in parallel, despite all formulations remaining within the hydrophilic range generally considered favorable for cell adhesion (30–57°; approximately 20–80°) [82,83]. Matrix stability at higher MC contents is a more likely explanation, consistent with the concentration-dependent gelation behavior of MC [84]. This interpretation is further supported by the swelling and degradation data (Figure 9a,b), which showed a delayed onset of degradation in the higher-MC, HA-containing scaffolds, indicative of greater matrix stability.

By day 7, the superior viability of the HA-containing scaffolds suggests that HA-related surface properties become increasingly influential once the initial adaptation phase is complete. Together with the cell-adhesive RGD motifs provided by gelatin and the improved surface hydrophilicity of HA-containing scaffolds and likely favors adsorption of adhesion-mediating serum proteins (Figure 7), which likely promote sustained cell attachment and proliferation [85,86,87,88].

Taken together, the temporary decline observed at day 3 for the lower-MC formulations is most consistent with an early cell-adaptation phase rather than inherent cytotoxicity, since all groups rebounded and surpassed the TCP control within four days, consistent with the transient lag phase commonly reported in three-dimensional culture systems [89,90,91]. This pattern of an initial deficit relative to conventional monolayer culture followed by a later crossover, where three-dimensional culture systems ultimately support cell numbers exceeding those on planar controls, has similarly been reported for other 3D biomaterial platforms [92]. By day 7, HA incorporation appeared to support sustained proliferation, potentially through its wettability-enhancing surface chemistry and intrinsic bioactivity.

#### 3.8.1. Cell Attachment and Migration (DAPI/Phalloidin Staining)

hBM-MSC behavior varied over time and with scaffold composition, as reflected in cytoskeletal organization. Consistent with the day-3 viability reduction described above (Figure 9c), phalloidin/DAPI staining at this time point showed predominantly rounded cells forming small clusters with little evidence of organized actin stress fibers (Figure 9d), indicative of an early attachment stage [93,94].

By day 7, the cellular phenotype had changed markedly. In parallel with the metabolic recovery described above, phalloidin staining revealed elongated, spindle-shaped cells with aligned actin filaments (Figure 9e). The emergence of organized stress fibers together with pronounced cell elongation is widely associated with stable cell adhesion and enhanced cell-material interactions [93,94]. These observations are consistent with the improved cytocompatibility observed by day 7 and with the reported role of HA in promoting cell adhesion, migration, and cytoskeletal organization through surface microtopography and calcium/phosphate ion release [86,87,95].

#### 3.8.2. Early Osteogenic Differentiation Assessed by ALP Activity

**Figure 10:**
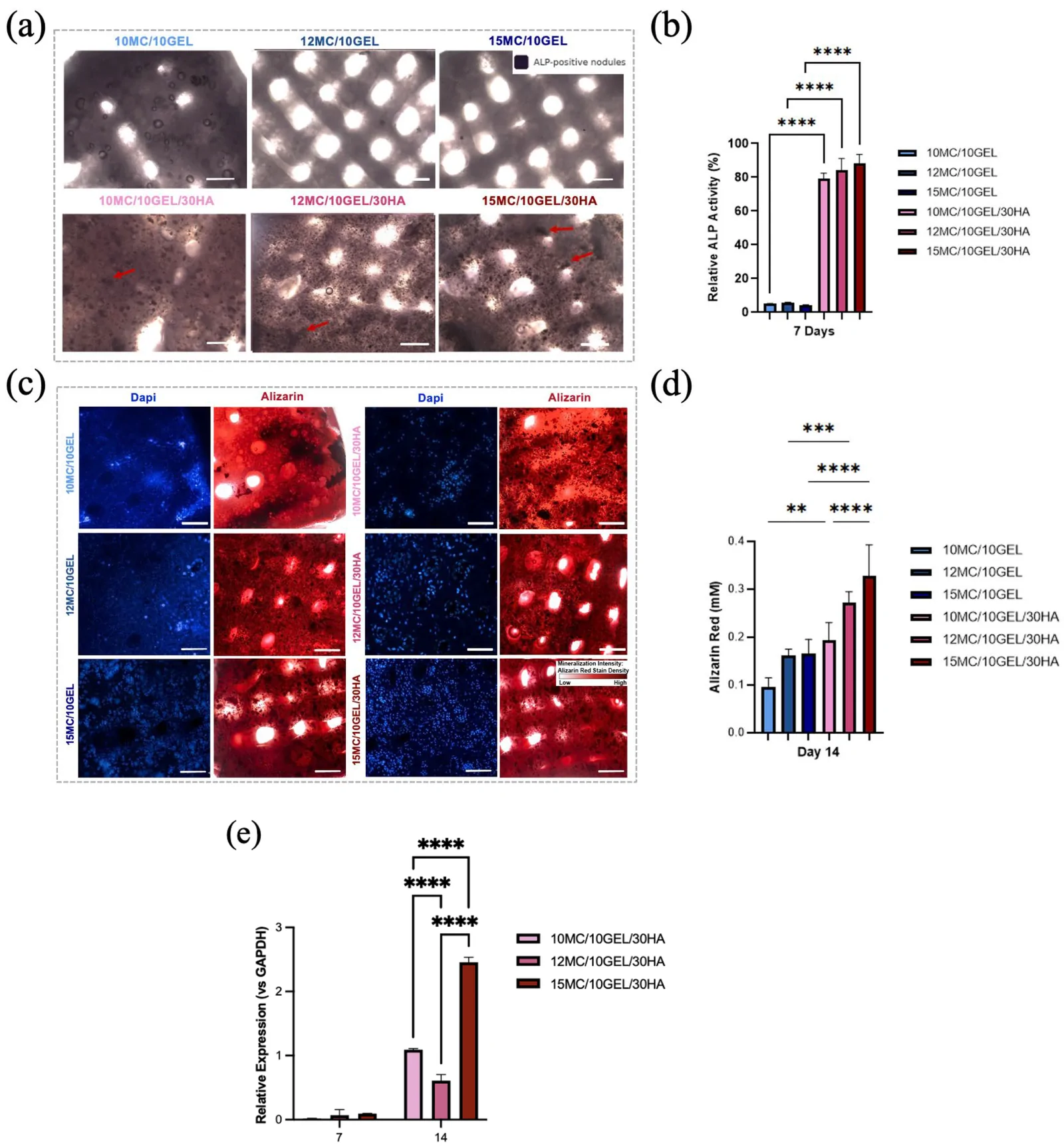
Osteogenic differentiation of hBM-MSCs on the MC/GEL and MC/GEL/HA scaffolds. (**a**) Representative brightfield images of BCIP/NBT histochemical staining showing ALP activity at day 7; red arrows indicate ALP-positive nodules. (**b**) Quantification of relative ALP activity (%) at day 7. (**c**) Representative fluorescence images of DAPI-stained nuclei (blue) and Alizarin Red-stained mineralized matrix (red) after 14 days of osteogenic induction. (**d**) Spectrophotometric quantification of extracted Alizarin Red at day 14. (**e**) OCN transcript expression (normalized to GAPDH) for the three HA-containing scaffolds at days 7 and 14. Data are mean ± SD; ** *p* < 0.01, *** *p* < 0.001, **** *p* < 0.0001. Scale bars = 500 µm; images acquired at 2× magnification; differences not indicated are non-significant.

ALP activity at day 7 was used to assess early osteogenic differentiation (Figure 10b). The six formulations segregated into two distinct groups. In the absence of HA, the HA-free constructs (10MC/10GEL, 12MC/10GEL and 15MC/10GEL) exhibited only near-background ALP activity, with relative values remaining below 10% of the reference level regardless of MC fraction. Incorporation of 30 wt % HA markedly increased ALP activity, with all HA-containing groups reaching 78–88% relative activity, representing more than a 10-fold increase compared with the corresponding HA-free scaffold (*p* <0.0001). No significant differences were observed among the HA-containing formulations, indicating that HA incorporation, rather than the MC/GEL ratio, was the primary determinant of the early osteogenic response.

This pattern was further supported histochemically by BCIP/NBT staining images (Figure 10a). HA-free scaffolds (top row) exhibited only sparse, weakly stained deposits, indicating limited early osteogenic activity. In contrast, HA-containing scaffolds (bottom row, red arrows) developed denser, more uniformly distributed dark purple/black ALP-positive nodules. The close agreement between the quantitative ALP measurements and the histochemical staining indicates that HA incorporation increased both the magnitude and the uniformity of ALP activity throughout the scaffolds. This is consistent with HA’s osteoconductive activity [96,97].

#### 3.8.3. Osteogenic Mineralization by Alizarin Red Staining and Quantification

After 14 days of osteogenic induction, mineralized matrix deposition was observed on all scaffolds, as distinct Alizarin Red-positive nodules (Figure 10c). HA apatite was deposited even in the HA-free formulations, indicating that the polymer matrix alone supported calcium-phosphate mineralization under osteogenic culture conditions. This effect may be partly due to the presence of gelatin, whose RGD-like sequences and charged residues support integrin-mediated adhesion and may create a favorable microenvironment for calcium phosphate nucleation [98,99,100].

Spectrophotometric quantification of the extracted dye revealed a graded, composition-dependent increase in mineral deposition (Figure 10d), with HA as the principal compositional driver. Incorporating 30 wt % HA raised the Alizarin Red signal, and the majority of HA versus HA-free contrasts reached significance (*p* < 0.01 to *p* < 0.0001). Calcium-phosphate mineralization peaked in 15MC/10GEL/30HA, approximately 2-fold above the 15MC/10GEL control, consistent with HA acting as a bone-mimetic template that lowers the energetic barrier to heterogeneous calcium phosphate deposition [101,102,103].

MC exerted an additional modulatory influence on mineralization. Increasing MC from 10% to 15% raised the Alizarin Red signal within each HA group, an effect most evident where HA was present, suggesting that MC modulates rather than independently drives the osteogenic influence of HA. This effect may instead reflect a direct physicochemical interaction between MC and the mineral phase rather than a swelling-mediated mechanism. Previous studies have suggested that methylcellulose can influence calcium phosphate mineralization by interacting with calcium and phosphate ions and modifying crystal growth behavior in HA-containing composites [88]. Other factors, such as MC-mediated dispersion of HA particles or resulting changes in protein and ion adsorption at the composite surface [104,105,106], were not directly examined here and are noted as candidate directions for future work rather than established mechanisms.

Fluorescence imaging supported these quantitative findings. DAPI-stained nuclei were uniformly distributed under all conditions, whereas the Alizarin Red signal progressed from sparse, diffuse staining in 10MC/10GEL to dense, merged nodules in the high-MC, HA-containing scaffolds (Figure 10c,d). Because DAPI and Alizarin Red images were acquired from the same field of view, co-registration showed that mineral deposition was concentrated in nuclei-rich regions rather than acellular areas, supporting a cell-associated mineralization process.

Comparison with the day-7 ALP results further suggests distinct roles for HA and MC during osteogenic differentiation. Whereas HA alone markedly increased early ALP activity, with little influence of MC content (Figure 10a,b), the later mineralization stage showed an additional MC-dependent increase superimposed on the HA effect (Figure 10c,d). This divergence suggests that HA predominantly drives early osteogenic commitment, whereas the MC/GEL ratio becomes influential only later, in regulating the extent of mineralization.

#### 3.8.4. Osteocalcin Gene Expression Analysis by RT-qPCR

Formulations with higher HA content were prioritized for OCN evaluation based on their favorable performance across earlier viability, ALP, and Alizarin Red assays. Osteocalcin, a non-collagenous matrix protein secreted by mature osteoblasts, is widely recognized as a molecular marker of late-stage osteogenic differentiation and matrix mineralization [107,108]. To assess this terminal stage of differentiation, OCN transcript levels (normalized to GAPDH) were measured in MC/GEL/HA scaffolds at days 7 and 14 (*n* = 3) (Figure 10e). At day 7, OCN expression remained low across all three formulations, with no significant differences observed between groups, consistent with cultures that had not yet entered the mineralization phase at this early stage. By day 14, the three formulations diverged markedly: the 15MC/10GEL/30HA scaffold showed the highest OCN expression (~2.45-fold).

The day-14 pattern is consistent with the expected late-stage increase in OCN expression during osteogenic differentiation [108]. It also aligns with the earlier viability, cytoskeletal findings, and mineralization results (Figure 9c–e and Figure 10c,d), where the 15MC/10GEL/30HA scaffold consistently showed the most favorable biological response among the HA-containing groups. These findings suggest that the more favorable early cell response may be associated with the enhanced late-stage osteogenic outcome, although the underlying mechanotransduction pathways (e.g., focal adhesion maturation or YAP/TAZ signaling) were not examined.

## 4. Conclusions

This work demonstrates that an EDC/NHS-crosslinked MC/GEL/HA ink can combine composition-dependent rheological control with reliable extrusion printability, mechanical performance, and osteoconductive bioactivity in a single bone-mimetic construct. MC content governed the viscoelastic and shear-thinning behavior of the ink, while HA acted as a composition-dependent reinforcing phase, particularly in formulations with lower MC content. The complementary roles of MC and HA were reflected in printed constructs with square-pore architectures, near-unity printability indices, and filament and pore dimensions within ranges considered favorable for cellular interaction and nutrient transport. Swelling, degradation, and pH measurements further indicated that HA incorporation modulated matrix stability and degradation behavior while sustaining a mildly alkaline microenvironment, a combination consistent with gradual structural evolution rather than premature structural failure. The progressive apatite layer detected by SEM, EDS, and FTIR after immersion in simulated body fluid confirmed that HA retained its intrinsic mineral-nucleating capacity even after being embedded within the crosslinked polymer network.

**Figure 11:**
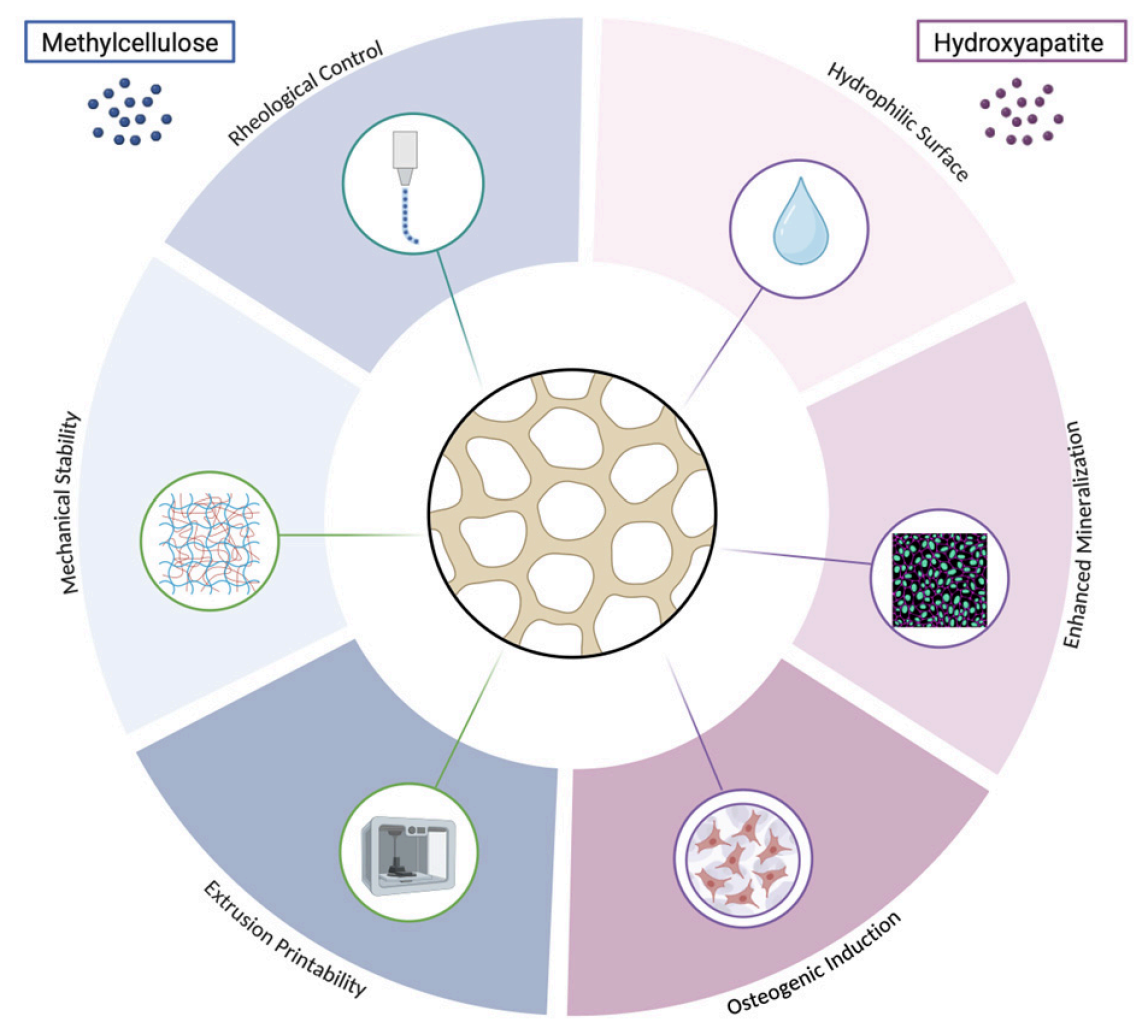
Schematic representation of the structural and functional properties of MC **(left side**) and HA (**right side**). Created in BioRender. Yuksel, C. (2026) https://BioRender.com/skhchow (Accessed on 26 July 2026).

These compositional effects were mirrored in the biological response of hBM-MSCs. Although hBM-MSCs showed a transient dip in metabolic activity during initial attachment, all scaffold groups demonstrated recovery and increased metabolic activity over time, accompanied by a shift from rounded, clustered cells to elongated morphologies with organized actin architecture. Rather than contributing redundantly, HA and MC appeared to influence distinct stages of osteogenic differentiation, with HA promoting early commitment and MC-dependent matrix properties contributing to later-stage mineral deposition and maturation. Among the evaluated formulations, 15MC/10GEL/30HA exhibited the most favorable balance of mechanical properties, matrix mineralization, and late-stage osteogenic maturation. Together, these findings indicate that balancing MC content and HA incorporation (Figure 11) provides a means of reconciling the competing demands of printability, structural integrity, and osteogenic performance within the range of formulations investigated. Overall, these results provide a rational design strategy for extrusion-printed osteogenic biomaterials suited to patient-specific reconstruction of irregular, non-weight-bearing bone defects, while underscoring the need for in vivo validation prior to clinical translation.

## Figures and Tables

**Table 1:** Percentages of MC/GEL/HA concentrations.

Methylcellulose (*w*/*v* %)	Gelatin (*w*/*v* %)	Hydroxyapatite (wt %)
10 *w*/*v* %	10 *w*/*v* %	30 wt %
12 *w*/*v* %	10 *w*/*v* %	30 wt %
15 *w*/*v* %	10 *w*/*v* %	30 wt %
10 *w*/*v* %	10 *w*/*v* %	Not included
12 *w*/*v* %	10 *w*/*v* %	Not included
15 *w*/*v* %	10 *w*/*v* %	Not included

**Table 2:** Primer sequences used for RT-qPCR.

Gene	Direction	Sequence (5′→3′)
OCN	Forward	GCAGCTTGGTGCACACCTAG
OCN	Reverse	GGAGCTGCTGTGACATCCAT
GAPDH	Forward	ATTTGGTCGTATTGGGCG
GAPDH	Reverse	TGGAAGATGGTGATGGGATT

## Data Availability

The original contributions presented in this study are included in the article. Further inquiries can be directed to the corresponding authors.

## Abbreviations

The following abbreviations are used in this manuscript:

MC	Methylcellulose
GEL	Gelatin
HA	Hydroxyapatite
EDC	1-Ethyl-3-(3-dimethylaminopropyl) carbodiimide
NHS	N-Hydroxysuccinimide
hBM-MSC	human Bone Marrow-derived Mesenchymal Stem Cell
RGD	Arginine-Glycine-Aspartate
SBF	Simulated Body Fluid
FTIR	Fourier-Transform Infrared Spectroscopy
SEM	Scanning Electron Microscopy
EDS	Energy Dispersive X-ray Spectroscopy
LVR	Linear Viscoelastic Region
G′	Storage modulus
G″	Loss modulus
η*	Complex viscosity
Pr	Printability index
PBS	Phosphate-Buffered Saline
W_0_/W_s_/Wd	Initial dry weight/Swollen (wet) weight/Dry weight
ALP	Alkaline Phosphatase
NBT/BCIP	Nitro Blue Tetrazolium/5-Bromo-4-Chloro-3-Indolyl Phosphate
DAPI	4′,6-Diamidino-2-Phenylindole
OCN	Osteocalcin
RT-qPCR	Reverse Transcription Quantitative Polymerase Chain Reaction
cDNA	Complementary DNA
GAPDH	Glyceraldehyde-3-Phosphate Dehydrogenase
Ct/ΔCt	Cycle threshold/Delta Cycle threshold
TCP	Tissue Culture Plastic
pHEMA	poly (2-Hydroxyethyl Methacrylate)
Ca/P	Calcium/Phosphorus ratio
ANOVA	Analysis of Variance
η^2^/partial η^2^	Eta-squared/Partial eta-squared
DMEM	Dulbecco’s Modified Eagle Medium
FBS	Fetal Bovine Serum
